# Longitudinal changes in the dental arches and soft tissue profile of untreated subjects with normal occlusion 

**DOI:** 10.1007/s00056-020-00221-x

**Published:** 2020-04-06

**Authors:** Sarah Stern, Hannah Finke, Marlon Strosinski, Silvia Mueller-Hagedorn, James A McNamara, Franka Stahl

**Affiliations:** 1 Department of Orthodontics, University Dental School, Strempelstraße 13, 18057 Rostock, Germany; 2 Department of Orthodontics and Pediatric Dentistry, School of Dentistry, University of Michigan, Ann Arbor, MI USA

**Keywords:** Dental development, Soft tissue profile, Children, Adolescents, Orthodontics, Gebissentwicklung, Weichteilprofil, Kinder, Jugendliche, Kieferorthopädie

## Abstract

**Aim:**

The aim of this study was to analyze dental and soft tissue profile development in children with normal occlusions to establish age- and gender-specific reference intervals for German children during their active growth period.

**Subjects and methods:**

The study group consisted of a sample of 31 untreated Caucasian subjects with normal occlusions. Dental casts were analyzed at four different stages of dentitional development. Extraoral profile photographs were available for 19 subjects at stages T2–T4. In these subjects 11 angular measurements and 14 indices were analyzed. Statistical comparisons of gender-specific differences were performed by Mann–Whitney U tests (*p* ≤ 0.05).

**Results:**

Upper and lower posterior and total arch perimeters were recorded to be significantly larger in male subjects until the late mixed dentition. Subsequently, there was a tendency toward larger dimensions in males for those parameters. Upper and lower intercanine, interpremolar and intermolar widths were significantly larger in males throughout the entire observation period. There were no statistically significant gender differences with regard to most angular measurements in the dental arches, including molar rotation, palatal volume, overbite, overjet and molar relationship at later dental stages.

**Conclusion:**

In untreated subjects with normal occlusion, dental arch and soft tissue parameters can be considered age-dependent. For some dental parameters, gender-specific differences were found that should be taken into consideration during diagnosis and treatment planning of growing children. The obtained longitudinal data of untreated children provide useful information for orthodontic diagnosis, treatment planning and future research projects.

## Abstract (de)

**Ziel:**

Ziel der Studie war es, bei Probanden mit normaler Okklusion die Gebissentwicklung und das Weichteilprofil zu analysieren und alters- und geschlechtsspezifische Referenzintervalle für deutsche Kinder während ihrer aktiven Wachstumsphase zu etablieren.

**Probanden und Methoden:**

Die Studiengruppe bestand aus 31 unbehandelten kaukasischen Probanden mit normaler Okklusion. Die Modelle wurden zu 4 unterschiedlichen Zeitpunkten der Gebissentwicklung analysiert. Extraorale Profilfotografien waren von 19 Probanden zu den Zeitpunkten T2–T4 verfügbar. Bei diesen Probanden wurden 11 Winkelmessungen und 14 Indizes analysiert. Der geschlechtsspezifische statistische Vergleich erfolgte mit Mann-Whitney-U-Tests (*p* ≤ 0,05).

**Ergebnisse:**

Bis zum späten Wechselgebiss waren der obere und untere hintere sowie der gesamte Zahnbogenumfang bei den männlichen Probanden signifikant größer. Daraus resultiert, dass auch danach noch ein Trend zu größeren Dimensionen bei den männlichen Probanden für diese Parameter vorlag. Die intercaninen und intermolaren Abstände waren im Ober- und Unterkiefer während des gesamten Beobachtungszeitraumes signifikant größer bei den männlichen Probanden. Zu späteren Zeitpunkten der Gebissentwicklung gab es keine statistisch signifikanten geschlechtsspezifischen Unterschiede in Bezug auf die meisten Winkelmessungen in den Zahnbögen, einschließlich Molarenrotation, Gaumenvolumen, Overbite, Overjet und Molarenbeziehung.

**Schlussfolgerung:**

Die Zahnbogenparameter und die Parameter des Weichteilprofils können bei unbehandelten Probanden mit normaler Okklusion als altersabhängig angesehen werden. Die für einige Zahnparameter gefundenen geschlechtsspezifischen Unterschiede sollten bei der Diagnostik und Behandlungsplanung von heranwachsenden Kindern berücksichtigt werden. Die erlangten Längsschnittdaten von unbehandelten Kindern bieten nützliche Informationen für die kieferorthopädische Diagnostik, Behandlungsplanung und für zukünftige Forschungsprojekte.

## Introduction

Orthodontic clinicians and researches benefit from the existence of gender- and age-specific standard values for specific populations for several reasons [13]. Standard values are of great help in diagnostics and treatment planning, especially in growing children in whom alveolar and dental changes occur continuously [10, 40]. Thus, knowledge of the dental arch changes that occur in untreated normal individuals during active growth years and beyond is especially important as it provides a baseline from which to plan orthodontic therapy [42, 33].

Furthermore, assessment of treatment outcome is more accurate by comparing natural growth changes in untreated subjects with normal occlusion rather than with controls displaying different types of malocclusion [34]. Therefore, several studies were carried out in the past to investigate longitudinal dental changes in untreated children and adolescents [4, 5, 7, 12, 13, 15, 20, 24, 27, 32, 34–36, 38–40]. However, only a few of them have described dental and soft tissue changes from the deciduous to the permanent dentition [5, 7, 8, 32, 38–40]. Selection criteria used for “standards” or “controls” in those studies are not uniform. They range from ideal occlusion [40] to good or acceptable occlusion [5, 7, 8, 12, 13, 24, 27, 32, 34–36, 39]. The inclusion criteria of the present study were rather strict [13, 17–19]. Only some studies have used comparable samples with a similar study design [8, 9, 15, 20, 24, 31, 32, 34, 39].

Until now, there have been no age- and gender-specific standard values for dental and soft tissue profile development that cover the period from the deciduous dentition until the permanent dentition in German Caucasian children. Therefore, the purpose of this investigation was to reintroduce data from a German Growth Study as a mean of analyzing dental arch and soft tissue profile dimensions in children and adolescents with normal occlusion. In greater detail, this study aims to establish first age- and gender-specific reference intervals for dental arch and soft tissue profile development for German juveniles and adolescents throughout dentitional development, and to examine gender differences during those age periods. From those data a first orientation of age- and gender-specific differences as well as reference intervals for several dental parameters at all dental stages can be taken. Those can be used for diagnosis and treatment planning in orthodontics.

## Subjects and methods

### Subjects

Ethical approval for this study was obtained from an Ethical Committee of a Medical Faculty in Germany (registration no. A 2018-0226). Parental consent was obtained prior to the study for all subjects included in this project. The dental casts for the present study were collected from a German Growth Study, which was initiated in 1959 by Prof. Dr. U. Klink-Heckmann in Germany. From 102 healthy subjects, dental impressions were taken every half year from birth until 2 years of age. Subsequently, dental impressions were taken annually until the age of 17 and in 17 subjects used in the present study until the age of 26. In addition, extraoral and intraoral photographs were obtained at every appointment during which dental impressions were taken. It must be mentioned that today it would be virtually impossible to obtain this type of longitudinal study material from healthy subjects who presented no obvious need for orthodontic treatment. More detailed information about this cohort was published by Stahl de Castrillon et al. in 2013 [13].

For the present study, the following inclusion criteria for defining a study group of untreated subjects with normal occlusion were applied: no orthodontic treatment throughout dentitional development, distocclusion of less than 2 mm in the deciduous canine region and bilateral flush terminal plane of deciduous molars in the deciduous and mixed dentitions, bilateral Class I or unilateral Class II molar relationship of less than 2 mm in the permanent dentition, fully developed deciduous, mixed and permanent dentitions with no missing teeth and no space loss due to early extraction or decay of deciduous teeth, only rotation of a single tooth, overjet of less than 3 mm, crowding of less than 2 mm in both dental arches, spacing in the frontal region of less than 2 mm in permanent dentition, midline deviation of less than 1 mm, and overbite of less than one third of coverage of lower incisors.

Thirty one subjects (16 males and 15 females) matched the inclusion criteria. Subjects were then matched according to subsequent dental stages and gender: T1—deciduous dentition (all deciduous teeth fully erupted), T2—early mixed dentition (all permanent incisors and first molars fully erupted, presence of deciduous canines and molars), T3—late mixed dentition (ongoing eruption of permanent canines and/or premolars), T4—permanent dentition (all permanent teeth fully erupted with the exception of third molars). For 21 subjects two or more sets of models were available for these developmental stages. The details of our cohort’s distribution for model analysis are shown in Table 1.

**Table 1:** Gender- and age-specific distribution of subjects for digital model analysis (age in years)

Dental stage	Male				Female			
	*n*	$\overline{x}$	Min	Max	*n*	$\overline{x}$	Min	Max
T1	16	3.5	2.5	3.8	14	3.6	3.1	4.6
T2	13	8.9	7.4	10.4	9	8.6	8.2	9.4
T3	14	10.7	9.2	12.2	12	10.4	8.5	12.1
T4	11	15.4	12.2	21.4	11	14.7	12.5	20.5

*T1* primary dentition, *T2* early mixed dentition, *T3* late mixed dentition, *T4* permanent dentition

For 19 of the selected subjects, black-and-white extraoral photographs of the soft tissue profile were available. These photographs were used for the soft tissue analysis at stages T2–T4. As the number of extraoral photographs was limited at certain dental stages (*n* = 7), we have chosen to report those data in Table 2 for illustrative purposes only.

**Table 2:** Gender- and age-specific distribution of subjects for soft tissue analysis (age in years)

Dental stage	Male				Female			
	*n*	$\overline{x}$	Min	Max	*n*	$\overline{x}$	Min	Max
T2	7	8.5	7.4	9.5	7	8.7	8.2	9.4
T3	9	10.4	9.2	12.2	10	10.6	9.1	12.1
T4	8	15.6	12.2	22.4	10	14.8	12.5	20.5

*T1* primary dentition, *T2* early mixed dentition, *T3* late mixed dentition, *T4* permanent dentition

### Three-dimensional dental model analysis

All dental casts were digitized by the same examiner (SS) using a high definition three-dimensional (3D) model scanner (Scanpoint 75T, Co. Elaboro, Schwerin, Germany). Software Geomagic® (Co. 3D Systems, Rock Hill, SC, USA) was then used to process the scanned models and register their occlusion. Three-dimensional model analysis was performed by a trained examiner (SS) using a customized model analysis tool by Cleft Dynamic® software (University of Rostock, Germany) [37].

**Fig. 1:**
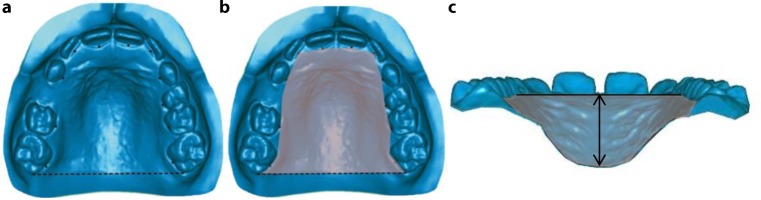
Assessment of palatal volume. Definition of palatal boundaries (gingival plane and distal plane) (**a**), palatal surface (**b**) and calculation of palatal volume (**c**)

The following measurements were obtained: anterior arch perimeter, posterior arch perimeter, and total arch perimeter for both arches [40]; upper and lower arch lengths [7, 40]; upper and lower intercanine, interpremolar and intermolar arch widths [20, 40] as well as the rotation of upper and lower first molars (defined as the angle between a straight line through the central fissure of the first permanent molar and the raphe median plane). Overbite [7], overjet [7] and molar relationship [6] were also analyzed. Palatal volume was measured three-dimensionally according to Primožič et al. [30]. First boundaries of the palate (gingival plane and distal plane) had to be defined. Then palatal volume could be calculated (Fig. 1).

### Soft tissue profile analysis

**Fig. 2:**
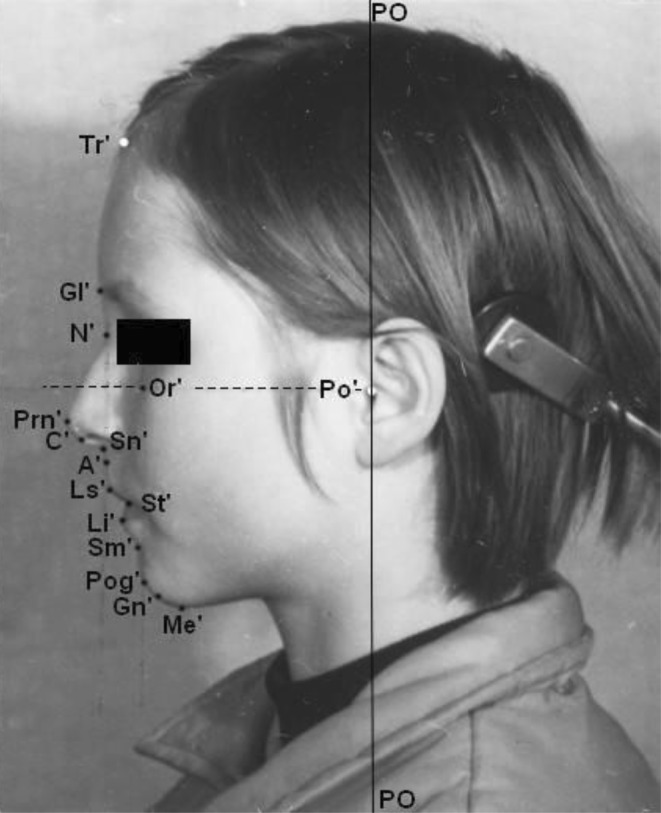
Reference points for soft tissue profile analysis

Extraoral photographs were digitized with a resolution of 600 dpi using high performance scanner Nexcan® (Co. Heidelberg, Germany). Customized analysis by the software fr–win® (Computer Konkret AG, Falkenstein, Germany) was used for soft tissue analysis. Measurements from different cephalometric analyses [2, 3, 5, 22, 25–27] and soft tissue analyses [1, 23, 28, 29, 36] were recorded. The reference points are shown in Fig. 2. Because it was not possible to determine magnification of extraoral photographs, only angular measurements were made and facial indices were calculated.

### Method error

From 10 randomly chosen subjects, extraoral photographs and dental casts were reanalyzed by one examiner (SS) to calculate method error by means of Dahlberg’s formula [14]. Mean differences in linear and angular measurements obtained through digital model analysis ranged between 0.1–0.9 mm and 1.6–2.9°, respectively. Mean differences in angular measurements of soft tissue profile analysis ranged from 0.3–3.6°.

### Statistical analysis

Descriptive statistics of dental arch and soft tissue profile parameters including the mean ($\overline{x}$) and standard deviation (SD) were calculated at 4 consecutive stages of dental development (T1–T4). Demographic homogeneity allowed for direct comparisons between males and females. It means that selection of subjects was realized by matching of different factors, e.g. gender, dental stage and presence of normal occlusion. The Shapiro–Wilk test revealed a lack of normal distribution for some variables. Therefore, nonparametric statistics by means of Mann–Whitney U tests were used to analyze gender differences at different dental stages (SPSS, Version 21, IBM, Armonk, NY, USA).

As we were aware of the fact that the limited number of subjects in the present study would lead to unstable results we constructed reference intervals by $\overline{\mathrm{x}}$± 1.96 × SD. They display 95% of all values by existence of normal distribution.

## Results

**Fig. 3:**
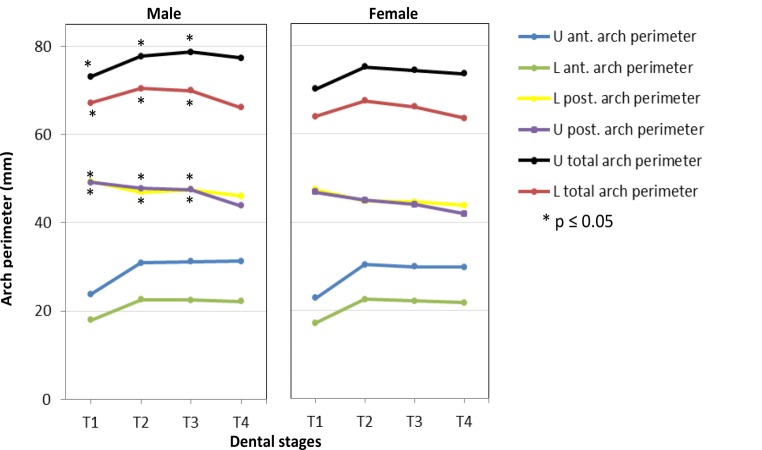
Results for gender-specific analysis of anterior (ant.), posterior (post.) and total arch perimeter. *T1* primary dentition, *T2* early mixed dentition, *T3* late mixed dentition, *T4* permanent dentition, *U* upper, *L* lower

**Fig. 4:**
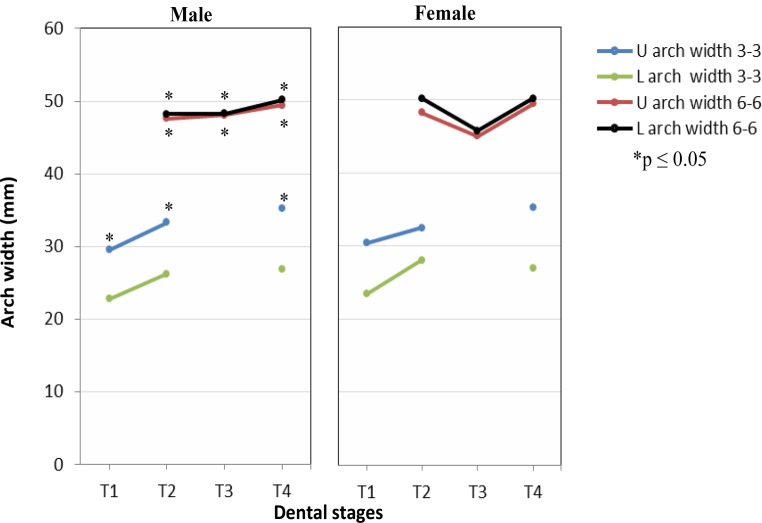
Results for gender-specific analysis of upper (U) and lower (L) intercanine and intermolar arch widths.* T1* primary dentition, *T2* early mixed dentition, *T3* late mixed dentition, *T4* permanent dentition

Means, standard deviations and reference intervals of dental arch parameters in untreated males and females with normal occlusion at consecutive dental stages T1–T4 are illustrated in Table 3. The corresponding soft tissue profile measurements at dental stages T2–T4 are shown in Table 4. Results being statistically significant for gender-specific analysis of dental arch parameters are displayed in Figs. 3 and 4.

**Table 3:** Means ($\overline{x}$), standard deviations (SD), reference intervals (RI) and gender-specific differences (*p ≤ 0.05) of dental arch parameters in untreated males and females with normal occlusion at consecutive dental stages T1 (primary dentition), T2 (early mixed dentition), T3 (late mixed dentition), T4 (permanent dentition)

		T1			T2			T3			T4		
	Dental arch parameter	$\overline{x}$	SD	RI	$\overline{x}$	SD	RI	$\overline{x}$	SD	RI	$\overline{x}$	SD	RI
Male	U ant. arch perimeter (mm)	23.8	1.6	20.7–26.9	30.9	1.2	28.6–33.1	31.2	1.9	28.6–33.1	31.2	2.7	26.0–36.4
	L ant. arch perimeter (mm)	18.0	0.8	16.4–9.6	22.9	0.8	21.0–24.2	22.5	1.1	21.0–24.2	22.2	1.0	20.3–24.1
	U post. arch perimeter (mm)	49.3*	2.1	45.3–53.4	46.9*	1.9	43.2–50.5	47.5*	3.3	43.2–50.5	46.1	3.6	39.0–53.2
	L post. arch perimeter (mm)	49.1*	2.3	44.6–53.7	47.8*	2.1	43.7–51.9	47.5*	3.0	43.7–51.9	43.9	2.8	38.4–49.4
	U total arch perimeter (mm)	73.1*	3.3	66.6–137.8	77.8*	2.6	72.7–82.9	78.7*	4.5	72.7–82.9	77.3	3.5	70.4–84.16
	L total arch perimeter (mm)	67.1*	2.9	61.4–72.8	70.4*	2.6	65.3–75.5	70.0*	3.5	65.3–75.5	66.1	3.7	58.8–73.4
	U arch width 3‑3 (mm)	29.5*	1.2	27.1–32.0	33.3*	1.7	30.0–36.6	–	–	30.0–36.6	35.3	1.9	31.6–39.0
	L arch width 3‑3 (mm)	22.8	1.5	19.8–25.8	26.3*	2.4	21.5–31.0	–	–	21.5–31.0	26.9*	1.8	23.3–30.5
	U arch width 4‑4 (mm)	–	–	–	–	–	–	–	–	–	37.4*	2.5	32.4–42.3
	L arch width 4‑4 (mm)	–	–	–	–	–	–	–	–	–	36.4*	2.3	31.9–40.9
	U arch width 5 5 (mm)	39.5*	2.0	35.6–43.4	–	–	–	–	–	–	43.3	2.0	39.4–47.2
	L arch width 5‑5 (mm)	39.5*	2.0	35.6–43.5	–	–	–	–	–	–	42.8	2.1	38.8–46.8
Male	U arch width 6‑6 (mm)	–	–	–	47.6*	2.1	43.5–51.6	48.1*	2.1	43.5–51.6	49.4*	1.6	46.3–52.6
	L arch width 6‑6 (mm)	–	–	–	48.2*	2.1	44.1–52.3	48.3*	2.3	44.1–52.3	50.2*	1.8	46.7–53.6
	U arch length (mm)	27.5	1.6	24.3–30.7	38.3	1.7	35.1–41.6	38.7	2.4	35.1–41.6	46.8	3.2	40.6–53.0
	L arch length (mm)	24.5*	1.5	21.7–27.4	35.0	1.4	32.2–37.7	35.1	2.1	32.2–37.7	42.9	2.7	37.6–48.3
	Molar rotation 16 (°)	–	–	–	16.9	2.8	11.5–22.4	16.4*	2.1	11.5–22.4	15.9	2.4	11.3–20.5
	Molar rotation 26 (°)	–	–	–	16.9	3.3	10.3–23.4	16.0	2.5	10.3–23.4	17.3	7.0	3.6–30.9
	Molar rotation 36 (°)	–	–	–	19.8	4.4	11.1–28.4	18.9	3.1	11.1–28.4	20.0	2.8	14.5–25.4
	Molar rotation 46 (°)	–	–	–	18.8	4.9	9.1–28.4	17.7	3.8	9.1–28.4	19.3*	2.2	14.9–23.7
	Palatal volume (mm^3^)	3021.0	410.9	2215.7–3826.4	6268.6	794.2	4712.0–7825.1	6261.6	1199.3	4712.0–7825.1	10218.9	1478.7	7320.7–13117.1
	Overbite (mm)	1.1	1.3	−1.3–3.6	1.4	2.0	−2.5–5.3	2.2	1.3	−2.5–5.3	2.2	1.5	−0.7–5.2
	Overjet (mm)	2.1	1.0	0.1–4.1	2.0	0.4	1.2–2.8	2.1	0.8	1.2–2.8	1.9	0.7	0.5–3.3
	Molar relationship right (mm)	0.0	0.8	−1.5–1.5	1.0	1.2	−1.3–3.2	1.4	1.6	−1.3–3.2	3.1	0.8	1.6–4.7
	Molar relationship left (mm)	0.3	0.4	−0.5–1.1	1.4	0.9	−0.4–3.3	1.3	1.7	−0.4–3.3	3.2	0.5	2.2–4.3
Female	U ant. arch perimeter (mm)	22.8	1.6	19.8–25.9	30.4	1.7	27.0–33.8	29.9	1.2	27.0–33.8	29.8	1.8	26.3–33.3
	L ant. arch perimeter (mm)	17.1	1.2	14.7–19.6	22.5	1.4	19.8–25.2	22.1	1.4	19.8–25.2	21.7	4.9	12.1–31.3
	U post. arch perimeter (mm)	47.4*	2.0	43.4–51.3	44.8*	1.6	41.7–47.8	44.6*	2.6	41.7–47.8	43.9	2.2	39.5–48.2
	L post. arch perimeter (mm)	46.8*	2.0	42.8–50.8	45.0*	2.1	41.0–49.0	44.0*	2.1	41.0–49.0	41.9	6.5	29.1–54.7
	U total arch perimeter (mm)	70.2*	3.2	63.9–76.5	75.2*	2.8	69.7–80.7	74.5*	3.2	69.7–80.7	73.7	3.6	66.6–80.8
	L total arch perimeter (mm)	64.0*	3.0	58.1–69.9	67.5*	2.8	–	66.1*	3.0	–	63.6	11.3	41.5–85.7
	U arch width 3‑3 (mm)	28.1*	1.5	25.2–31.0	31.0*	1.7	27.6–34.4	–	–	27.6–34.4	33.4*	1.4	30.7–36.2
	L arch width 3‑3 (mm)	21.8	1.2	19.4–24.2	25.7	1.3	23.2–28.2	–	–	23.2–28.2	25.8	1.3	23.3–28.3
	U arch width 4‑4 (mm)	–	–	–	–	–	–	–	–	–	34.4*	2.4	29.8–39.1
	L arch width 4‑4 (mm)	–	–	–	–	–	–	–	–	–	34.5*	1.9	30.9–38.2
	U arch width 5‑5 (mm)	37.7*	2.1	33.6–41.7	–	–	–	–	–	–	39.7*	2.3	35.2–44.3
	L arch width 5‑5 (mm)	38.0*	2.2	33.7–42.2	–	–	–	–	–	–	40.3*	2.3	35.7–44.8
Female	U arch width 6‑6 (mm)	–	–	–	45.0*	2.6	40.0–50.0	45.1*	2.4	40.0–50.0	45.5*	2.3	40.9–50.0
	L arch width 6‑6 (mm)	–	–	–	46.2*	2.5	41.3–51.1	45.8*	2.4	41.3–51.1	46.8*	2.4	42.1–51.6
	U arch length (mm)	26.4	1.8	22.8–30.0	37.3	2.2	33.0–41.5	37.2	2.1	33.0–41.5	45.6	2.7	40.3–50.9
	L arch length (mm)	23.0*	1.7	19.6–26.4	34.0	2.3	29.5–8.5	33.9	2.2	29.5–8.5	42.1	2.5	37.1–47.0
	Molar rotation 16 (°)	–	–	–	18.7	4.3	10.2–27.2	19.8*	5.3	10.2–27.2	17.5	2.4	12.8–22.2
	Molar rotation 26 (°)	–	–	–	18.2	4.8	8.7–27.7	18.7	5.5	8.7–27.7	16.9	2.7	11.6–22.2
	Molar rotation 36 (°)	–	–	–	20.6	5.2	10.4–30.8	21.8	5.4	10.4–30.8	22.5	4.3	14.1–30.8
	Molar rotation 46 (°)	–	–	–	21.6	6.1	9.6–33.5	20.9	4.8	9.6–33.5	23.3*	5.7	12.1–34.5
	Palatal volume (mm^³^)	2963.8	453.9	2074.2–3853.5	5905.2	656.1	4619.2–7191.2	6229.3	662.2	4619.2–7191.2	9573.5	1194.2	7232.9–11914.0
	Overbite (mm)	1.5	0.9	−0.3–3.3	2.1	1.0	0.2–4.1	2.1	2.1	0.2–4.1	2.4	1.9	−1.4–6.1
	Overjet (mm)	2.3	1.0	0.3–4.3	1.8	0.8	0.2–3.5	2.1	0.7	0.2–3.5	1.8	0.7	0.3–3.2
	Molar relationship right (mm)	0.1	0.7	−1.2–1.5	1.9	1.1	−0.3–4.1	1.8	1.1	−0.3–4.1	2.7	0.4	1.9–3.4
	Molar relationship left (mm)	0.3	0.4	−0.5–1.1	1.7	0.6	0.6–2.9	1.6	1.4	0.6–2.9	2.5	0.7	1.2–3.8

*U* upper, *L *lower, *ant*. anterior, *post.* posterior

**Table 4:** Means ($\overline{x}$), standard deviations (SD), reference intervals (RI) and gender-specific differences (*p ≤ 0.05) of soft tissue profile parameters in untreated males and females with normal occlusion at consecutive dental stages T2 (early mixed dentition), T3 (late mixed dentition), T4 (permanent dentition)

	Soft tissue profile parameters	T2			T3			T4		
		$\overline{x}$	SD	RI	$\overline{x}$	SD	RI	$\overline{x}$	SD	RI
Male	*Total profile*									
	Gl’-Prn’-Pog’ (°)	143.0	4.7	133.8–152.2	140.9	2.4	136.3–145.5	135.3	2.4	130.5–140.0
	Gl’-Sn’-Pog’ (°)	164.7	3.5	157.8–171.7	163.1	1.6	160.1–166.2	161.6	1.7	158.2–165.0
	PO-Sn’/[½ × (PO-N’ + PO-Pog’)] × 100 (%)	109.1	2.0	1.1–1.1	109.4	1.7	1.1–1.1	109.1	1.4	1.1–1.1
	(PO-Prn’/PO-N’) × 100 (%)	115.6	1.8	112.1–119.0	116.3	2.2	112.0–120.5	118.5	3.2	112.3–124.7
	(PO-Sn’/PO-N’) × 100 (%)	103.5	2.1	99.5–107.6	103.5	2.0	99.6–107.4	104.6	2.3	100.1–109.2
	(PO-B’/PO-N’) × 100 (%)	90.9	2.6	85.9–96.0	90.2	2.7	84.9–95.4	91.4	3.1	85.4–97.4
	*Lip profile*									
	C’-Sn’-Ls’ (°)	121.3	12.4	97.0–145.6	114.4	12.9	89.1–139.7	121.4	9.6	102.6–140.2
	Ls’-N’-Pog’ (°)	10.1	2.6	5.0–15.3	11.3*	1.9	122.6–154.1	10.0	2.2	118.8–145.0
	Ls’-N’-Li’ (°)	4.3	1.9	0.7–7.9	5.6	1.3	7.6–15.1	5.4	1.6	5.7–14.3
	Li’-N’-Pog’ (°)	5.9	1.6	2.8–8.9	5.7*	1.6	3.2–8.1	4.5	1.8	2.4–8.5
	FH/N’-Ls’ (°)	93.6	3.0	87.6–99.5	94.6	3.3	2.6–8.8	94.8	2.4	1.0–8.1
	FH/N’-Li’ (°)	89.3	1.8	85.7–92.8	89.0	2.4	88.2–101.0	89.3	2.0	90.2–99.4
	(PO-Ls’ / PO-Li’) × 100 (%)	104.4*	1.7	101.1–107.6	105.9	1.0	104.0–107.8	105.8	1.9	102.1–109.5
Male	*Chin prominence*									
	FH/N’-Pog (°)	83.4	1.9	79.8–87.1	83.3	2.7	78.1–88.5	84.8	3.0	79.0–90.6
	(PO-Pog’/PO-B’) × 100 (%)	98.6	1.6	95.5–101.8	99.1	2.4	94.4–103.8	100.4	2.3	95.9–104.8
	Li’-B’-Pog’ (°)	144.9	8.7	97.0–145.6	138.4	8.0	122.6–154.1	131.9	6.7	118.8–145.0
	*Vertical height relation*									
	(N’-Sn’/Sn’-Me’) × 100 (%)	67.7	7.0	54.0–81.5	72.0	8.0	56.3–87.7	79.8	8.1	64.1–95.6
	(Sn’-St’/St’-Me’) × 100 (%)	52.2	4.2	43.9–60.4	48.4	6.1	36.4–60.3	44.7	6.7	31.6–57.8
	(Tr’-N’/Tr’-Me’) × 100 (%)	38.4	2.6	33.3–43.5	38.6	2.3	34.1–43.1	34.7	3.1	28.6–40.8
	(N’-Sn’/Tr’-Me’) × 100 (%)	24.9	2.2	20.6–29.2	25.7	2.3	21.2–30.2	29.0	2.8	23.5–34.8
	(Sn’-Me’/Tr’-Me’) × 100 (%)	36.8	1.5	33.9–39.7	35.7	1.7	32.4–39.0	36.3	1.2	33.9–38.7
	(Sn’-St’/Sn’-Me’) × 100 (%)	34.5	1.6	31.4–37.6	32.6	2.9	26.9–38.1	30.9	3.3	24.4–37.4
	(St’-Me’/Sn’-Me’) × 100 (%)	65.7	1.7	62.4–69.0	67.6	3.1	61.5–73.7	69.2	3.3	62.7–75.7
	*Sagittal intermaxillary relationship*									
	FH/Ls’-Pog’ (°)	67.9	3.3	61.5–74.3	66.2	4.1	58.2–74.1	68.6	4.6	59.7–77.5
	(PO-Sn’/PO-B’) × 100 (%)	113.9	2.3	109.3–118.5	114.9	2.1	110.8–118.9	114.6	2.6	109.5–119.7
Female	*Total profile*									
	Gl’-Prn’-Pog’ (°)	140.2	4.1	132.2–148.2	139.2	3.3	132.7–145.6	135.6	3.2	113.8–126.3
	Gl’-Sn’-Pog’ (°)	163.2	4.4	154.6–171.7	164.1	3.7	156.8–171.4	136.3	3.9	100.1–110.3
	PO-Sn’/[½ × (PO-N’ + PO-Pog’)] × 100 (%)	109.8	3.6	1.0–1.2	108.5	2.9	1.1–1.1	108.9	2.0	1.1–1.1
	(PO-Prn’/PO-N’) × 100 (%)	117.4	4.1	109.5–125.4	116.5	3.3	109.9–123.0	120.0	3.2	113.8–126.3
	(PO-Sn’/PO-N’) × 100 (%)	104.3	4.1	96.3–112.4	103.1	2.7	97.9–108.4	105.2	2.6	100.1–110.3
	(PO-B’/PO-N’) × 100 (%)	91.7	4.7	82.5–100.9	90.8	4.5	82.0–99.6	93.2	3.5	86.3–100.1
	*Lip profile*									
	C’-Sn’-Ls’ (°)	117.6	7.2	122.4–170.2	113.8	9.6	105.4–186.4	111.4	10.8	117.8–156.0
	Ls’-N’-Pog’ (°)	10.2	2.5	5.3–15.2	8.8*	1.3	6.2–11.5	9.0	1.4	6.3–11.7
	Ls’-N’-Li’ (°)	5.5	1.3	3.0–8.0	5.2	1.3	2.6–7.8	5.3	1.3	2.8–7.7
	Li’-N’-Pog’ (°)	4.7	1.6	1.6–7.9	3.6*	0.9	1.9–5.4	3.8	1.1	1.7–5.9
	FH/N’-Ls’ (°)	94.3	4.8	84.9–103.7	92.6	3.4	86.0–99.2	95.0	2.5	90.1–99.9
	FH/N’-Li’ (°)	88.8	3.8	81.4–96.3	87.4	3.2	81.1–93.8	89.8	2.8	84.2–95.3
	(PO-Ls’/PO-Li’) × 100 (%)	106.2*	1.3	103.8–108.7	106.3	2.0	102.5–110.1	105.6	1.9	102.0–109.3
Female	*Chin prominence*									
	FH/N’-Pog (°)	84.1	3.1	78.1–90.1	83.8	2.9	78.1–89.4	86.0	2.8	80.6–91.4
	(PO-Pog’/PO-B’) × 100 (%)	98.3	2.5	93.3–103.2	99.1	2.0	95.1–103.1	100.4	1.2	98.0–102.8
	Li’-B’-Pog’ (°)	146.3	12.2	122.4–170.2	145.9	20.7	105.4–186.4	136.9	9.8	117.8–156.0
	*Vertical height relation*									
	(N’-Sn’/Sn’-Me’) × 100 (%)	73.7	8.7	56.7–90.8	75.2	6.1	95.0–132.6	76.9	8.2	60.8–93.0
	(Sn’-St’/St’-Me’) × 100 (%)	53.4	4.7	44.3–62.6	58.8	26.5	105.4–186.4	48.3	5.4	37.7–58.9
	(Tr’-N’/Tr’-Me’) × 100 (%)	39.0	2.4	34.3–43.7	36.8	3.3	30.3–43.3	36.8	3.3	30.3–43.3
	(N’-Sn’/Tr’-Me’) × 100 (%)	25.8	1.5	22.9–28.8	27.1	2.0	23.2–31.0	27.9	2.7	22.6–33.2
	(Sn’-Me’/Tr’-Me’) × 100 (%)	35.3	2.9	29.6–41.0	36.1	2.2	31.8–40.4	36.4	2.4	31.7–41.1
	(Sn’-St’/Sn’-Me’) × 100 (%)	34.8	1.9	31.1–38.5	35.9	7.7	20.8–51.0	32.4	2.3	27.9–36.9
	(St’-Me’/Sn’-Me’) × 100 (%)	65.2	1.9	61.5–68.9	64.2	7.7	49.1–79.3	68.3	3.6	61.2–75.4
	*Sagittal intermaxillary relationship*									
	FH/Ls’-Pog’ (°)	68.2	3.5	61.4–75.0	68.9	2.9	63.2–74.6	71.0	4.4	62.5–79.6
	(PO-Sn’/PO-B’) × 100 (%)	113.9	3.1	107.9–119.9	113.7	3.8	106.2–121.2	113.0	2.5	108.0–118.0

### Three-dimensional model analysis

Anterior arch perimeter increased from the deciduous to the mixed dentition period due to eruption of permanent incisors in both arches; these measurements then remained relatively constant during the rest of observation period. In contrast, posterior arch perimeter decreased in both arches during dentitional development. Interestingly total arch perimeter increased significantly between the deciduous and permanent dentition period in the maxilla (males +4.2 mm, females +3.5 mm), whereas it decreased in the mandible during the entire observation period (males −1.0 mm, females −0.4 mm). These findings can be explained by size differences of deciduous molars in both arches and a greater mesial movement of first and second permanent molars in the mandible. In the deciduous and mixed dentitions, gender differences were observed only for posterior and total arch perimeters in both arches, displaying significantly greater dimensions in male subjects (Fig. 3).

From the deciduous to the permanent dentition, intercanine distance increased significantly in the maxilla (males +5.8 mm, females +5.3 mm) and in the mandible (males +4.1 mm, females +4.0 mm). Intermolar distances increased slightly in the upper (males 1.8 mm, females 0.5 mm) and lower jaw (males 2.0 mm, females 0.6 mm). Again, significantly greater dimensions for arch widths were determined in male subjects (Fig. 4).

As expected, upper and lower arch lengths increased significantly during the total observation period in males and females, with no statistically significant gender differences.

Molar rotation was maintained during the observation period in males and females in both arches. No gender differences were recorded for molar rotation.

Palatal volume increased significantly during the observation period in both genders (+120%). There were no gender differences recorded for palatal volume measurements.

No significant differences in overbite and overjet were recorded throughout the entire observation period. There also were no significant gender differences recorded for those parameters.

Molar relationship increased bilaterally in males (+3.0 mm) and females (+2.5 mm) displaying more mesial movement of the first permanent molars in the lower jaw to achieve a Class I relationship. This difference was not gender-specific.

### Soft tissue profile analysis

#### Overall profile

Gl’-Prn’-Pog’ angle decreased, while Gl’-Sn’-Pog’ angle remained stable, displaying nasal development during the observation period in both genders. These findings were supported by the increments of PO-Prn’/PO-N’ index and stability of PO-Sn’/PO-N’ index from the mixed to the permanent dentition.

#### Lip profile

Most angular measurements and indices representing changes in lip profile remained stable throughout the entire observation period. Only nasolabial angle (C’-Sn’-Ls’ angle) and mentolabial fold angle (Li’-B’-Pog’ angle) decreased slightly more in females until permanent dentition. However, those differences were not gender-specific.

#### Chin prominence

Chin prominence increased slightly more in females from the mixed to the permanent dentition. This increase in prominence was shown by an increase of the FH/N’-Pog’ angle and the PO-Pog’/PO-B’ index as well as by a decrease of the Li’-B’-Pog’ angle. Again, no statistically significant gender differences were noted in those parameters.

#### Vertical height relation

During the observation period, upper facial height (Tr’-N’/Tr’-Me’ index) decreased, while midfacial height (N’-Sn’/Tr’-Me’ index) increased. Lower facial height (Sn’-Me’/Tr’-Me’ index) remained stable. No gender differences were reported for any of these indices.

#### Sagittal intermaxillary relationship

Sagittal intermaxillary relationship remained stable throughout the entire observation period in both genders, as indicated by no changes in the FH/Ls’-Pog’ angle or the PO-Sn’/PO-B’ index. No statistically significant gender differences were observed for sagittal intermaxillary relationship.

## Discussion

As no age- and gender-specific standard values exist so far for German Caucasian children the intention of this paper was to present first reference intervals which can be used as a guideline for orthodontic diagnosis and treatment planning. To do so the current study analyzed material from a unique European sample of longitudinal records from untreated subjects with normal occlusion and well-balanced faces. All subjects were of German origin and resided in the northeastern part of Germany. The inclusion criteria for participating subjects were rather strict. Dental models were collected in most subjects from birth until the age of 17 years. In addition, extraoral photographs were taken for some of the participating subjects. As data sets taken from extraoral photographs were incomplete, the conclusions drawn from these results should be handled with caution. However, we have chosen to report those data for illustrative purposes.

As we were aware of the fact that the limited number of subjects in the present study would lead to unstable results selection of subjects was realized by matching of different factors like dental stage, gender and presence of normal occlusion. Besides descriptive statistics reference intervals were constructed as they maintain 95% of all values by existence of normal distribution. As the results of this study aimed to give a first orientation for diagnostic purposes in orthodontics further studies with a similar study design and a greater sample size need to be carried out in order to verify the present data.

As all longitudinal studies face the problem of missing data, we decided not to fill in those missing data with records from additional subjects, as occurred in the Bolton-Brush Growth Study [11]. Instead we have chosen to report our data without substituting the records of other individuals for the missing time points. To reduce methodological error of 3D model and soft tissue analysis, we had a single investigator to analyze all dental casts and photographs. She had been trained previously by an experienced researcher (MS).

Calculation of method error revealed high reproducibility of landmark identification on dental casts and extraoral photographs for most measurements, with the notable exception of molar rotation. Because of larger fillings in these teeth, it was more difficult to identify precise landmarks in some subjects. In addition, because the magnification factor for analyzing dimensions in extraoral photographs was not available, only angles were measured and indices calculated.

Males and females showed an increase in anterior dental arch perimeter during dentitional development [8, 40]. The decrease in posterior and total arch perimeters, especially in the mandible, is caused by losing leeway space and the mesial movement of permanent molars [40]. These findings support the results of previous studies that have shown that dental arch length is decreasing constantly until adult age [21, 40, 41]. Intercanine and intermolar widths increased in both arches during dentitional development.

Most increments of upper and lower anterior dental arch widths occurred in the deciduous and early mixed dentition period. Finally, 60% and 85% of natural increase in intercanine distances occurred between the deciduous and early mixed dentition period in both jaws. This observation might lead to expanding earlier in children with crowded/collapsed arches than recommended in the current treatment protocols.

As it was one of the intentions of this study to present three-dimensional data of interest, palatal volume was measured at different stages of dental development. Palatal volume increased continuously from deciduous to permanent dentition and was recorded to be gender-specific. These observations confirm previous findings by Yang et al. [43].

In the current study, there was only a minimum increase in overbite (+1.0 mm) throughout the entire observation period, findings that are similar to the results of other studies [4, 20, 34]. Unlike other studies [16, 20, 40, 41], however, we found no longitudinal changes in the amount of overjet. As expected in subjects with normal occlusion, the molar relationship increased from the deciduous to permanent dentition, confirming results of other investigations [6].

To describe gender differences at subsequent dental developmental stages, mean gender differences were compared (Tables 3 and 4). Gender-specific differences for dental arch parameters were identified only in deciduous and mixed dentitions for posterior and total dental arch perimeter, whereas for upper intercanine width as well as upper and lower intermolar distances gender differences were found throughout the entire observation period. It is obvious that dentofacial dimensions grow with advancing age. For all linear measurements, male values were larger than female values. It should be mentioned that due to the limited number of male and female subjects some gender-specific differences might not be detectable. Therefore the presented data have to be interpreted with caution. Of course, adding more subjects to the study group would have been very favorable. Unfortunately, this kind of study material is difficult to gather and no more untreated subjects with normal occlusion from this cohort were available.

Finally, the investigated soft tissue profile changes in this study should be interpreted with caution as of incomplete data sets. Out of 31 subjects with normal occlusion and well-balanced faces, extraoral photographs were available in 19 subjects. In addition, some reference points, e.g., Tr’, were difficult to identify as they were covered on some photographs by the subject’s hair. In contrast to Bishara et al. [5], we found no gender-specific differences in soft tissue profiles of subjects with normal occlusion and permanent dentition. A previous cephalometric study which was conducted on the same study material revealed a counter clockwise rotation in males and females. There were also no statistically significant gender differences found on vertical angle measurements [13]. Only the nasolabial angle and the mentolabial fold angle decreased slightly more in females until permanent dentition, whereas chin prominence increased more in females from the mixed to the permanent dentition. However, significant gender differences were not identified for any of these parameters. As expected in subjects with well-balanced faces, facial proportions in the vertical and sagittal dimension remained stable throughout the entire observation period in both genders.

## Conclusion

The results of this study of untreated subjects with normal occlusion show that dental development can be considered age- and gender-dependent. These observations should be considered when diagnosis and treatment planning of children and adolescents in orthodontics is undertaken. However, due to the limited number of subjects used to establish age- and gender-specific reference intervals, the presented results have to be interpreted with caution. More longitudinal studies with a similar study design and greater sample size should be carried out in the future in order to verify the established data.

For therapeutic purposes, several interesting results from this study can be taken into consideration. Thus, especially in patients where anterior crowding in the lower jaw is seen early (during the eruption of the permanent incisors), the first permanent molars should be held in place (e.g., transpalatal arch, lingual holding arch) or even distalized before the eruption of permanent canines and premolars begins. Early intervention will help to reduce the naturally occurring decrease of total arch perimeter during later stages of dental development. Furthermore major natural increases in intercanine distances occurring very early in dentitional development should be considered and used intentionally especially in the treatment of children with more severe frontal crowding or narrow apical base in the frontal region.
